# Supplementary material for: Redox-Active Monolayers Self-Assembled on Gold Electrodes—Effect of Their Structures on Electrochemical Parameters and DNA Sensing Ability
Source: Molecules. 2020 Jan 30;25(3):607. doi: 10.3390/molecules25030607 (PMC7037899; doi:10.3390/molecules25030607)
Supplement: Supplementary file 1 [file molecules-25-00607-s001.pdf]

Supplementary Information

Redox-active monolayers self-assembled on gold electrodes - effect of their structures on electrochemical parameters and DNA sensing ability

Kamila Malecka [1], Shalini Menon [2], Gopal Palla [1], Krishnapillai Girish Kumar [2], Mathias Daniels[3], Wim Dehaen [3], Hanna Radecka [1] and Jerzy Radecki [1,*]

[1] Institute of Animal Reproduction and Food Research, Polish Academy of Sciences, Tuwima 10, 10-748 Olsztyn, Poland;

[2] Department of Applied Chemistry, Cochin University of Science and Technology, Kochi-682022, Kerala, India;

[3] Molecular Design and Synthesis, Department of Chemistry, KU Leuven, Leuven Chem&Tech, Celestijnenlaan 200F, B-3001 Leuven, Belgium

* Correspondence: j.radecki@pan.olsztyn.pl

Content

1. Synthesis of TPY-NHS
2. Procedure for calculation of electron transfer coefficients α and electron transfer rate constants k [s^{-1}].
3. **Figure S1.** Representative cyclovoltammograms obtained for the gold electrode modified with:
   A) TPY/Co(II)/TPY/EA (solid line) and TPY/Co(II)/TPY/ssDNA (dashed line)
   B) TPY/Cu(II)/TPY/EA (solid line) and TPY/Cu(II)/TPY/ssDNA (dashed line)
   C) DPM/Co(II)/TPY /EA (solid line) and DPM/Co(II)/TPY/ssDNA (dashed line)
   D) DPM/Cu(II)/TPY/EA (solid line) and) DPM/Cu(II)/TPY/DNA (dashed line)
   Buffer conditions: PBS pH 7.4, Scan Rate: 100 mV/s
   EA – NH_2-CH_2-CH_2-OH
4. **Figure S2.** Representative Osteryoung square-wave voltammograms obtained for the gold electrode modified with:
   A) TPY/Co(II)/TPY/EA (solid line) and TPY/Co(II)/TPY/ssDNA (dashed line)
   B) TPY/Cu(II)/TPY/EA (solid line) and TPY/Cu(II)/TPY/ssDNA (dashed line)
   C) DPM/Co(II)/TPY /EA (solid line) and DPM/Co(II)/TPY/ssDNA (dashed line)
   D) DPM/Cu(II)/TPY/EA (solid line) and) DPM/Cu(II)/TPY/DNA (dashed line)
   Buffer conditions: PBS pH 7.4
5. **Figure S3.** An example of the CV curves obtained for the gold electrode modified with: (A) TPY/Co(II)/TPY/EA, and (C) TPY/Co(II)/TPY/ssDNA
   B,D) plot of (●, Ipa) anodic and (■, Ipc) cathodic peak current against potential scan rate; Scan rates: 0.050–1.0 V/s.
6. **Figure S4.** An example of the CV curves obtained for the gold electrode modified with: (A) TPY/Cu(II)/TPY/EA, and (C) TPY/Cu(II)/TPY/DNA
   B,D) plot of (●, Ipa) anodic and (■, Ipc) cathodic peak current against potential scan rate; Scan rates: 0.050–1.0 V/s.
7. **Figure S5.** An example of the CV curves obtained for the gold electrode modified with: (A) DPM/Co(II)/TPY/EA, and (C) DPM/Co(II)/TPY/ssDNA
   B,D) plot of (●, Ipa) anodic and (■, Ipc) cathodic peak current against potential scan rate; Scan rates: 0.050–1.0 V/s.
8. **Figure S6.** An example of the CV curves obtained for the gold electrode modified with: (A) DPM/Cu(II)/TPY/EA, and (C) DPM/Cu(II)/TPY/ssDNA
   B,D) plot of (●, Ipa) anodic and (■, Ipc) cathodic peak current against potential scan rate; Scan rates: 0.050–1.0 V/s.
9. **Table S1:** Comparison of electrochemical genosensors presented with those already published.

1. Synthesis Terpy NHS ester

All reagents and solvents were purchased from commercial sources and used as received unless noted otherwise. Reactions were carried out in standard glassware with Teflon-coated magnetic stirring bars on a magnetic stirrer under nitrogen atmosphere. NMR spectra were measured on a Bruker Avance 300 apparatus. Shift values are expressed in ppm relative to tetramethylsilane (^{1}H, 0) or solvent signal (^{13}C{^{1}H}, DMSO-d6: 39.52). Melting point was determined on a Mettler-Toledo DSC822 instrument, using a heating rate of 0.5 °C.min^{-1} under helium atmosphere. HRMS spectra were acquired on a quadrupole orthogonal acceleration time-of-flight mass spectrometer (Synapt G2 HDMS, Waters, Milford, MA). Samples were infused at 3µL/min and spectra were obtained in positive (or: negative) ionisation mode with a resolution of 15000 (FWHM) using leucine enkephalin as lock mass.

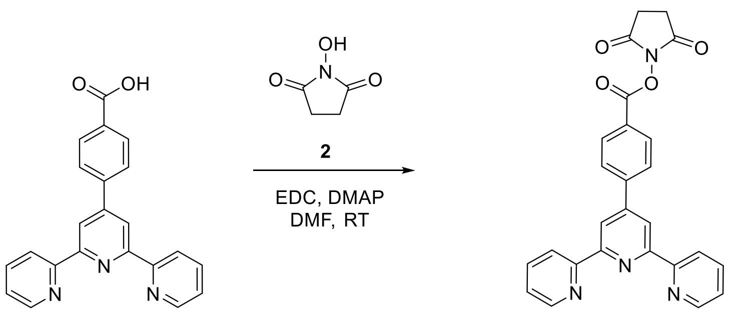

Compound **1** was prepared according to literature procedure **[1].**

To a suspension of compound **1** (3.53 g, 10 mmol), *N*-Hydroxysuccinimide (1.27 g, 11 mmol) and DMAP (0.12 g, 1 mmol) in DMF (100 ml) stirring at room temperature was added EDC (2.9 g, 15 mmol) and the reactiom mixture was stirred untill forming a clear solution and TLC indicated complete conversion of compound **1**. The solvent was removed under reduced pressure and DCM (50 ml) was added. Compound 2,5-dioxopyrrolidin-1-yl 4-([2,2':6',2''-terpyridin]-4'-yl)benzoate (2 g, 4.44 mmol, 44.4 % yield) precipitated as a white amorphous solid. HRMS (ES^{+}): calculated for $C_{26}H_{19}N_4O_4^{+}$ [M+H]$^{+}$: 451.1401; found: 451.1397. ^{1}H NMR (300 MHz, DMSO-d6) δ 8.82 – 8.72 (m, 4H), 8.70 – 8.63 (m, 2H), 8.34 – 8.23 (m, 2H), 8.23 – 8.15 (m, 2H), 8.10 – 7.98 (m, 2H), 7.59 – 7.49 (m, 2H), 2.94 (s, 4H). ^{13}C{^{1}H} NMR (75 MHz, DMSO-d6) δ 170.18, 155.89, 154.60, 149.29, 147.64, 143.84, 137.41, 130.90, 127.98, 124.97, 124.59, 120.91, 118.11, 25.54.

References:

1. Martínez, M.; Carranza, M. P.; Massaguer, A.; Santos, L.; Organero, J. A.; Aliende, C.; De Llorens, R.; Ng-Choi, I.; Feliu, L.; Planas, M.; Rodríguez, A.M.; . Manzano, B.R.; Espino G.; Jalón, F.A. Synthesis and biological evaluation of Ru(II) and Pt(II) complexes bearing carboxyl groups as potential anticancer targeted drugs. *Inorg. Chem.* **2017**, *56* (22), 13679–13696. DOI: 10.1021/acs.inorgchem.7b01178.

2. Procedure for calculation of α and k [s^{-1}]

To determine the electron transfer coefficient α, the peak potential E_p is plotted *vs.* log ν [1]. E_{pa} and E_{pc} are plotted separately in this way to give two branches. The slope of the line is given in *Equation 1*:

$$slope = -\frac{2.3RT}{\alpha nF} \qquad (1)$$

Determining the x-intercepts of the lines for the anodic and the cathodic branches provides ν_a and ν_c, respectively, values that are used in *Equation 2* to determine the electron transfer rate constant k [2]:

$$k = \frac{\alpha nF v_c}{RT} = \frac{(1-\alpha)nF v_a}{RT} \qquad (2)$$

where: n is the number of electrons involved in the oxidation or reduction process, F is the Faraday constant, R is the ideal gas constant, T is the temperature.

References:

1. Laviron E.J., General expression of the linear potential sweep voltammogram in the case of diffusionless electrochemical systems. *J. Electroanal. Chem.* **1979**, *101*,19–28.
2. Eckermann, A.L.; Feld, D.J.; Shaw, J.A.; Meade, T.J. Electrochemistry of redox-active self-assembled monolayers. *Coord. Chem. Rev.* **2010**, *254*, 1769-1802. DOI: 10.1016/j.ccr.2009.12.023

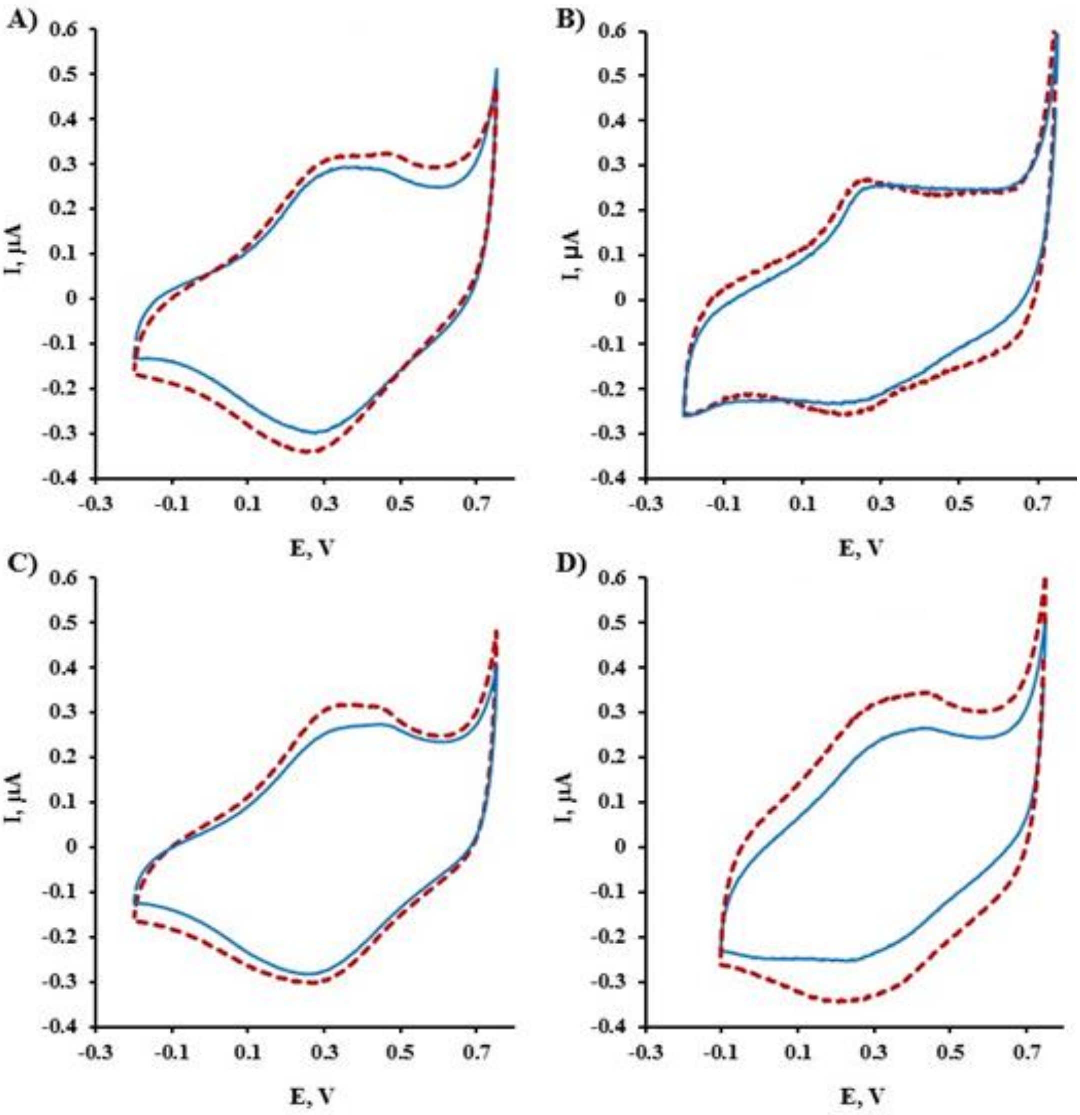

Figure S1. Representative cyclovoltammograms obtained for the gold electrode modified with:

A) TPY/Co(II)/TPY/EA (solid line) and TPY/Co(II)/TPY/ssDNA (dashed line)

B) TPY/Cu(II)/TPY/EA (solid line) and TPY/Cu(II)/TPY/ssDNA (dashed line)

C) DPM/Co(II)/TPY /EA (solid line) and DPM/Co(II)/TPY/ssDNA (dashed line)

D) DPM/Cu(II)/TPY/EA (solid line) and) DPM/Cu(II)/TPY/DNA (dashed line)

Buffer conditions: PBS pH 7.4, Scan Rate: 100 mV/s

EA – NH_2-CH_2-CH_2-OH

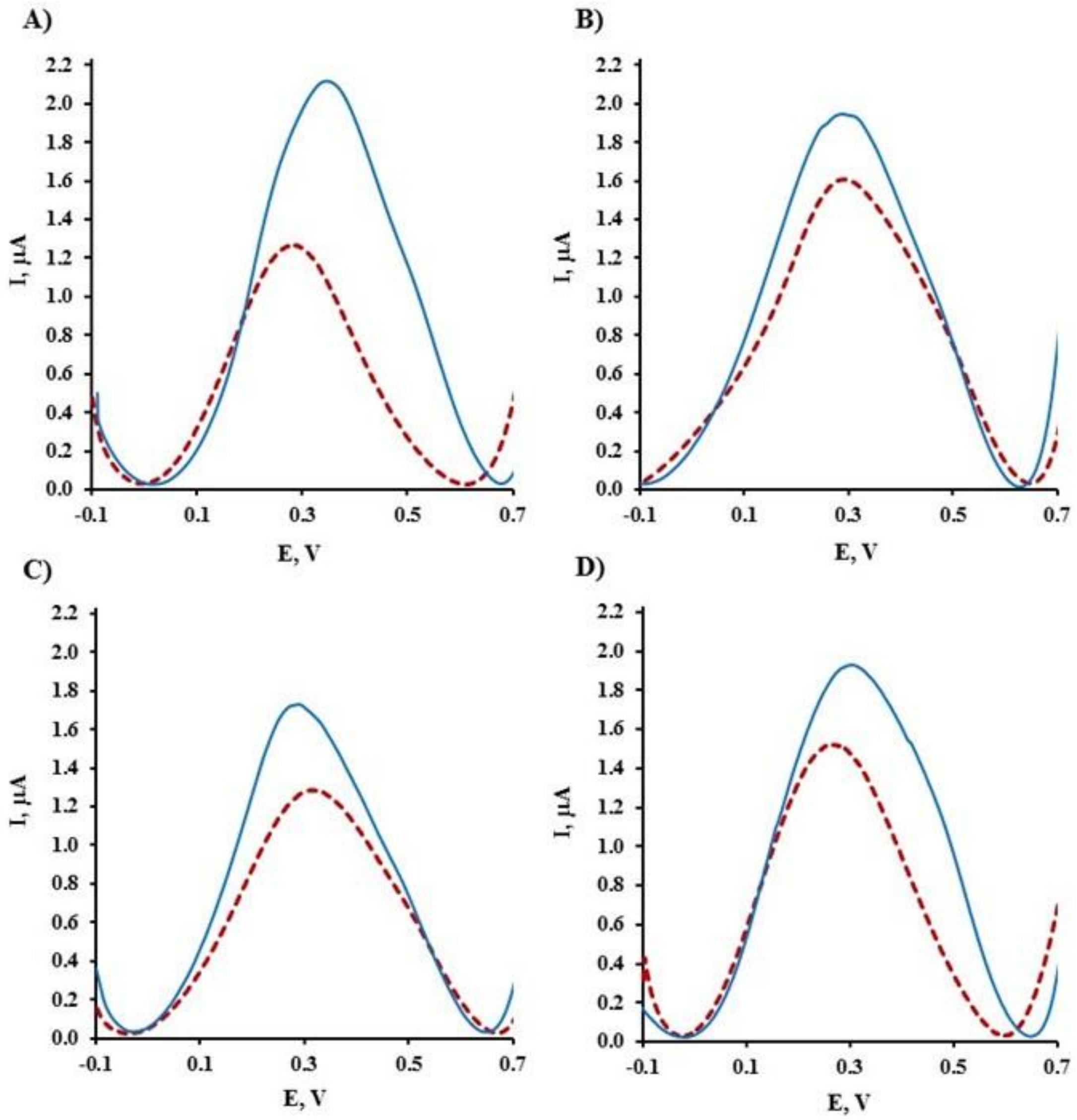

Figure S2. Representative Osteryoung square-wave voltammograms obtained for the gold electrode modified with:

A) TPY/Co(II)/TPY/EA (solid line) and TPY/Co(II)/TPY/ssDNA (dashed line)

B) TPY/Cu(II)/TPY/EA (solid line) and TPY/Cu(II)/TPY/ssDNA (dashed line)

C) DPM/Co(II)/TPY /EA (solid line) and DPM/Co(II)/TPY/ssDNA (dashed line)

D) DPM/Cu(II)/TPY/EA (solid line) and) DPM/Cu(II)/TPY/DNA (dashed line)

Buffer conditions: PBS pH 7.4

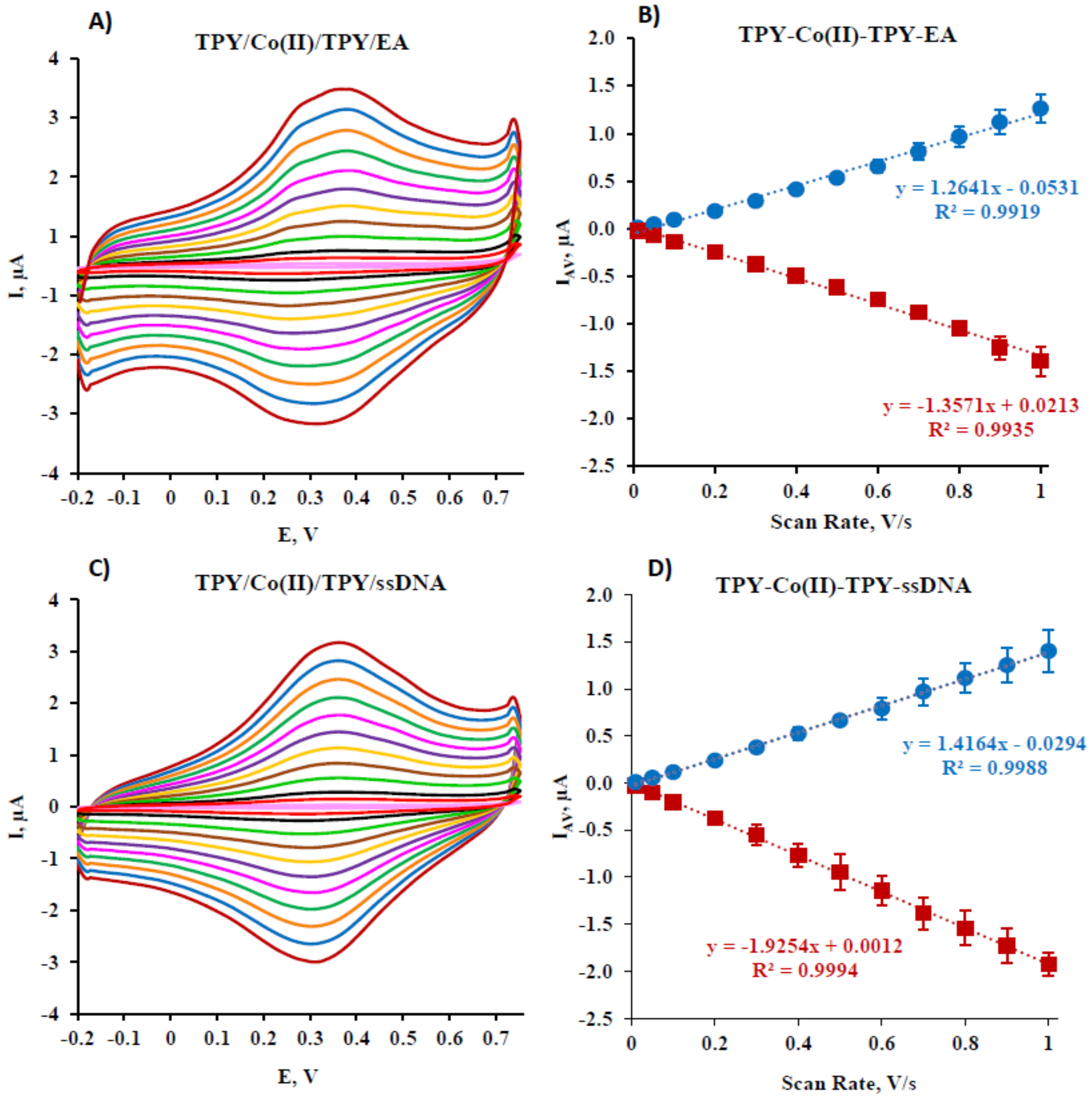

Figure S3. An example of the CV curves obtained for the gold electrode modified with: (A) TPY/Co(II)/TPY/EA, and (C) TPY/Co(II)/TPY/ssDNA
B,D) plot of (●, I_{pa}) anodic and (■, I_{pc}) cathodic peak current against potential scan rate; Scan rates: 0.050–1.0 V/s.

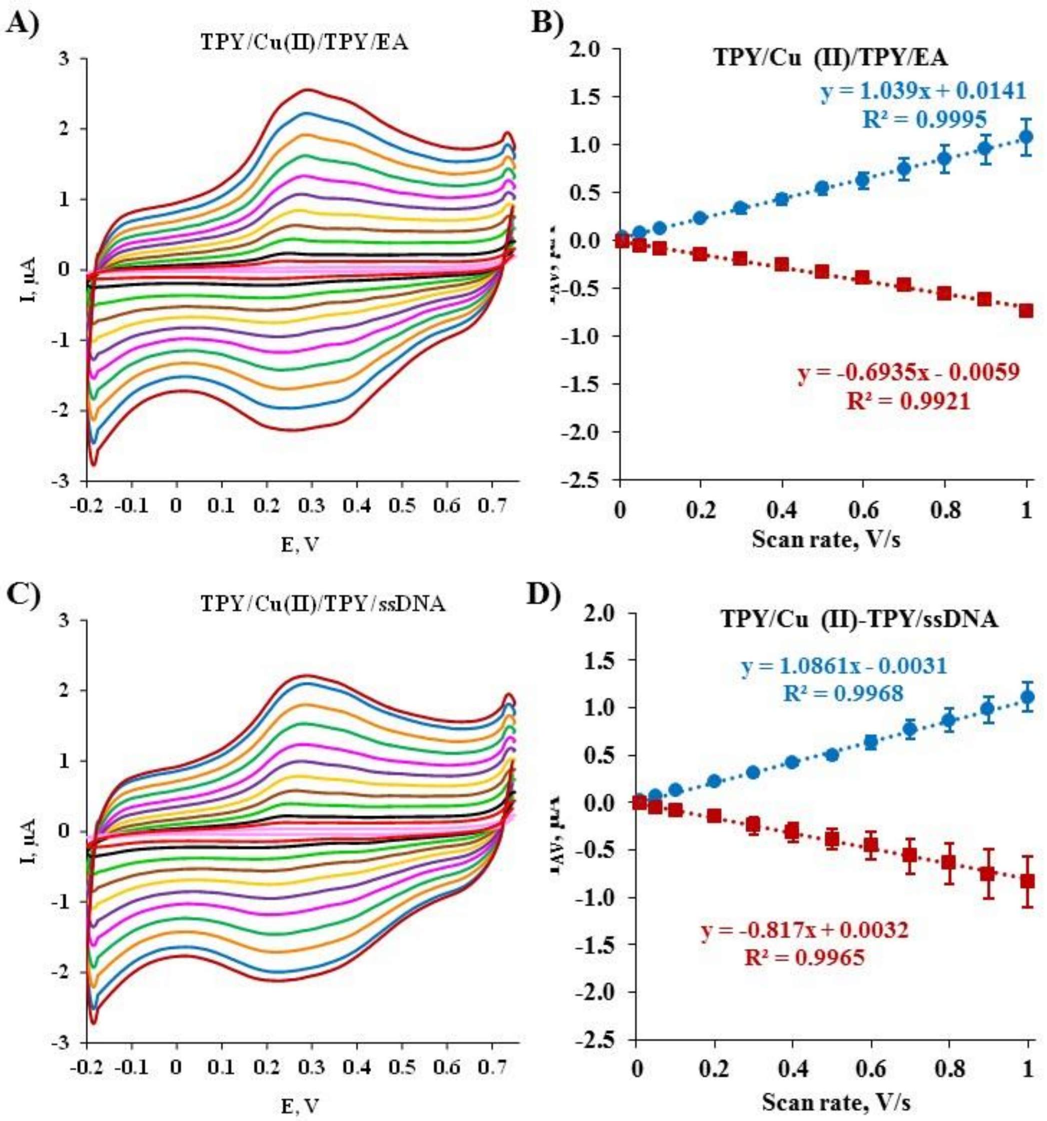

Figure S4. An example of the CV curves obtained for the gold electrode modified with: (A) TPY/Cu(II)/TPY/EA, and (C) TPY/Cu(II)/TPY/DNA
B,D) plot of (●, I_{pa}) anodic and (■, I_{pc}) cathodic peak current against potential scan rate; Scan rates: 0.050–1.0 V/s.

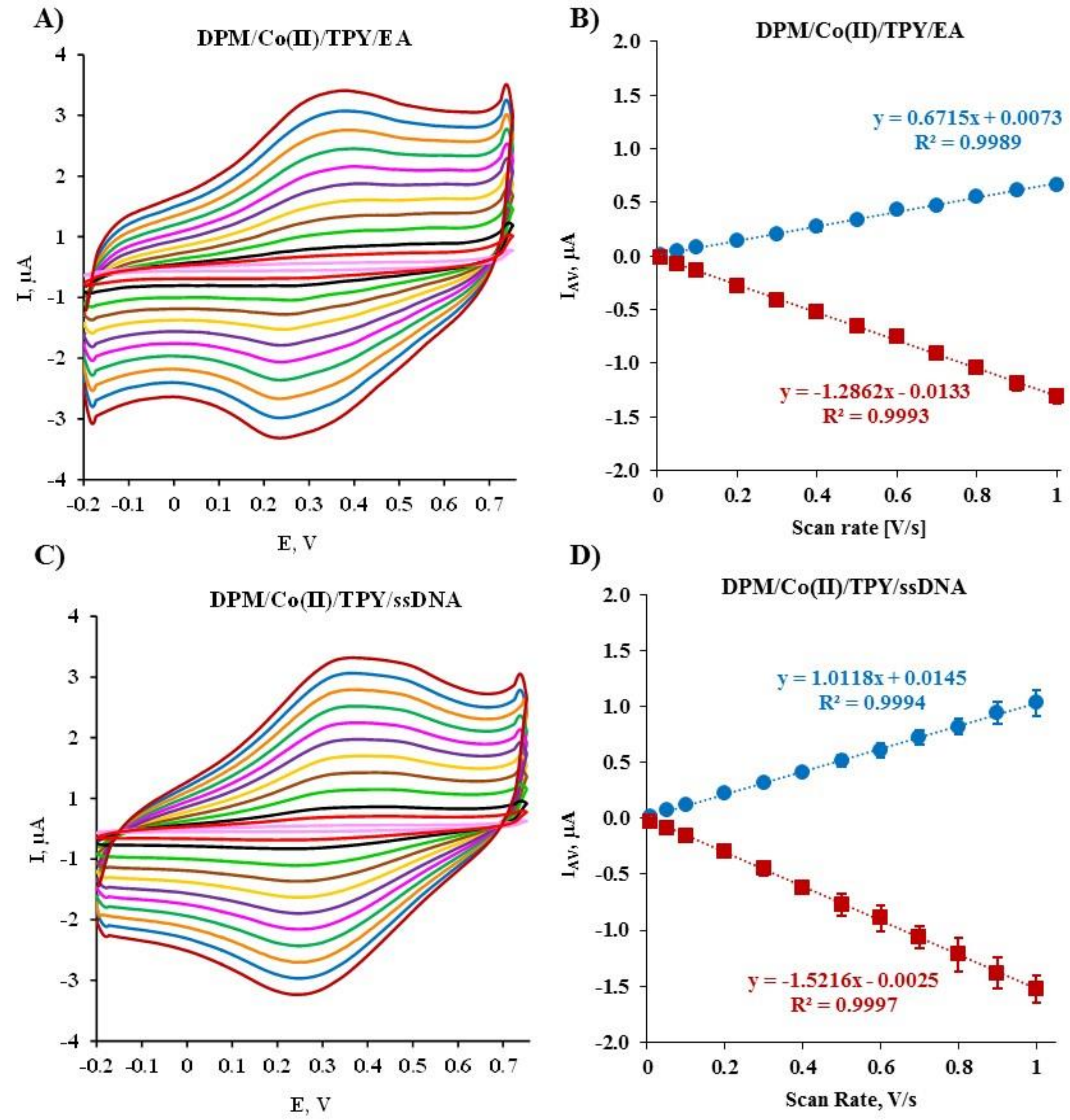

Figure S5. An example of the CV curves obtained for the gold electrode modified with: (A) DPM/Co(II)/TPY/EA, and (C) DPM/Co(II)/TPY/ssDNA
B,D) plot of (●, I_{pa}) anodic and (■, I_{pc}) cathodic peak current against potential scan rate; Scan rates: 0.050–1.0 V/s.

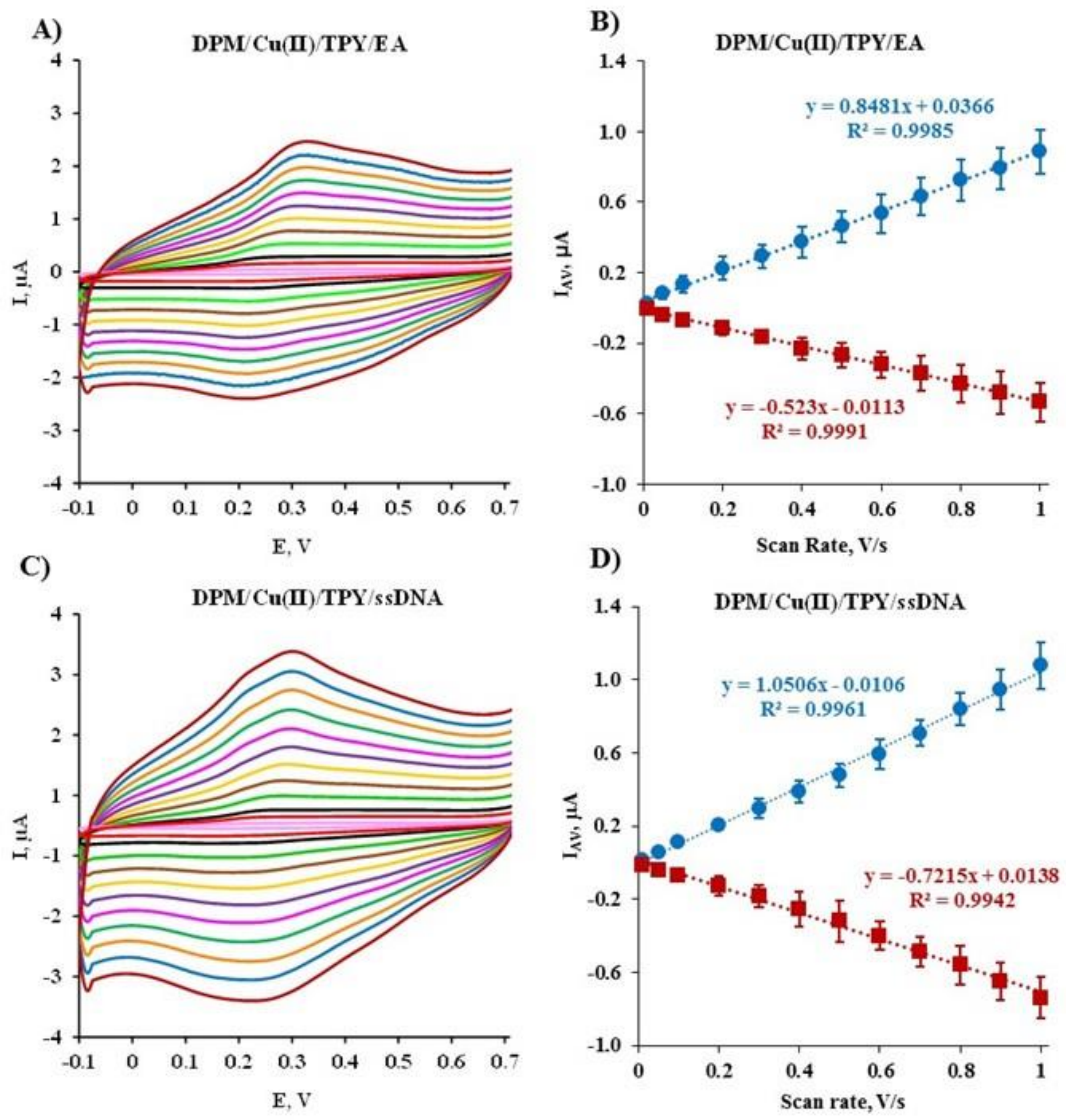

Figure S6. An example of the CV curves obtained for the gold electrode modified with: (A) DPM/Cu(II)/TPY/EA, and (C) DPM/Cu(II)/TPY/ssDNA

B,D) plot of (●, I_{pa}) anodic and (■, I_{pc}) cathodic peak current against potential scan rate; Scan rates: 0.050–1.0 V/s.

Table S1: Comparison of electrochemical genosensors presented with those already published.

Electrode modification	Measuring technique	Target	Detection limit [M]	References
Au/SH-ssDNA-MB + SH-ssDNA-Fc + MCH /MCH	OSWV	20 mer ssDNA	18-21×10^{-9}	[1]
Au/CoP-ssDNA/MCH		20 mer ssDNA	10^{-14}	[2]
Au/MPA/ NH_2-3-iron bis(dicarbollide)-ssDNA		20 mer ssDNA and 181 mer dsDNA	3×10^{-17}<br>8×10^{-17}	[3]
SPCE/CHT/PNA-AQ		14 mer ssDNA	4.0 × 10^{-9}	[4]
Au/MBT+DPM-SH/Co(II)/DPM-COOH/NH_2-ssDNA<br>Au/MBT+DPM-SH/Cu(II)/DPM-COOH/NH_2-ssDNA		20 mer ssDNA	1.28×10^{-12}<br>1.39×10^{-12}	[5]
Au/AET/Phen-Epoxy/ Fe(III)/(Phen-Epoxy)$_2$/NH_2-ssDNA		20 mer ssDNA and ca. 280 mer RNA	7.3×10^{-11}<br>8.7×10^{-13}	[6]
Au/SH-ssDNA-Fc/MCH+MB-primer		31 mer ssDNA	2.8×10^{-14} M	[7]
Au/SH-G-DNA1/ G-DNA2 + Hem + t-ssDNA	DPV	30 mer ssDNA	5.4×10^{-14}	[8]
GCE/depAuNPs/SH-ssDNA/MCH/t-ssDNA/ AgNCs		18 mer ssDNA	1.62 × 10^{-16}	[9]
Au/CA/ GA/ Fc-PAMAM G1/GA/ ssDNA<br>Au/CA/ GA/ Fc-PAMAM G2 /GA/ ssDNA<br>Au/CA/ GA/ Fc-PAMAM G3 / GA/ ssDNA		24 mer ssDNA	3.8 × 10^{-10}<br>9.2 × 10^{-10}<br>6.6 × 10^{-10}	[10]
Au/MBT+AHT/TPY-NHS/Co(II)/ TPY-NHS /NH_2-ssDNA<br>Au/MBT+ AHT/TPY-NHS /Cu(II)/ TPY-NHS /NH_2-ssDNA<br>Au/MBT+DPM-SH/Co(II)/ TPY-NHS /NH_2-ssDNA<br>Au/MBT+DPM-SH/Cu(II)/ TPY-NHS /NH_2-ssDNA	OSWV	20 mer ssDNA	2.13 × 10^{-15}<br>1.58 × 10^{-15}<br>5.43 × 10^{-15}<br>1.01 × 10^{-15}	This work

Abbreviations: Au – gold electrode, ssDNA – single stranded DNA, MB – Methylene Blue, Fc – ferrocene, MCH – 6-mercaptohexan-1-ol, OSWV – Osteryoung Square Wave Voltammetry, CoP – Cobalt Porphyrin, MPA – mercaptopropionic acid, SPCE – Screen-printed carbon electrode, CHT – chitosan, PNA – Peptide Nucleic Acid, AQ – antraquinone, MBT – 4-mercapto-1-butanol; DPM – dipyrromethene; AET - 2-aminoethanethiol hydrochloride, Phen-Epoxy – 5,6-epoxy-5,6-dihydro-[1.10]-phenanthroline, MB-Primer – 8mer ssDNA modified with MB, G-DNA – G-quadruplex-DNA, Hem – hemin, DPV – Differential Pulse Voltammetry, GCE – glassy carbon electrode, depAuNPs – deposited gold nanoparticles, t-ssDNA – ssDNA target, AgNCs – silver nanoclusters, CA- cysteamine; Fc-PAMAM G1, G2, G3 – three different ferrocene-cored poly(amidoamine) dendrimers generations; GA – glutaraldehyde; AHT – 6-amino-1-hexanethiol; TPY-NHS – terpirydine with NHS.

References

1. Grabowska, I.; Malecka, K.; Stachyra, A.; Góra-Sochacka, A.; Sirko, A.; Zagórski-Ostoja, W.; Radecka, H.; Radecki, J. Single electrode genosensor for simultaneous determination of sequences encoding hemagglutinin and neuraminidase of avian influenza virus type H5N1. *Anal. Chem.* **2013**, *85*, 10167-10173. DOI: 10.1021/ac401547h.
2. Grabowska, I.; Singleton, D.G.; Stachyra, A.; Góra-Sochacka, A.; Sirko, A.; Zagórski-Ostoja, W.; Radecka, H.; Stulz, E.; Radecki, J. A highly sensitive electrochemical genosensor based on Co-porphyrin-labelled DNA. *Chem. Commun.* **2014**, *50*, 4196-4199. DOI: 10.1039/c4cc00172a.
3. Grabowska, I.; Stachyra, A.; Góra-Sochacka, A.; Sirko, A.; Olejniczak, A.B.; Leśnikowski, Z.J.; Radecka, H.; Radecki, J. DNA probe modified with 3-iron bis(dicarbollide) for electrochemical determination of DNA sequence of Avian Influenza Virus H5N1. *Biosens. Bioelectron.* **2014**, *51*, 170-176. DOI: 10.1016/j.bios.2013.07.026.
4. Jampasa, S.; Wonsawat, W.; Rodthongkum, N.; Siangproh, W.; Yanatatasaneejit, P.; Vilaivan, T.; Chailapakul, O. Electrochemical detection of human papillomavirus DNA type 16 using a pyrrolidinyl peptide nucleic acid probe immobilized on screen-printed carbon electrodes. *Biosens. Bioelectron.* **2014**, *54*, 428-434. DOI: 10.1016/j.bios.2013.11.023.
5. Kurzątkowska, K.; Sirko, A.; Zagórski-Ostoja, W.; Dehaen, W.; Radecka, H.; Radecki J. Electrochemical label-free and reagentless genosensor based on an ion barrier switch-off system for DNA sequence-specific detection of the Avian Influenza Virus. *Anal. Chem.* **2015**, *87*, 9702–9709. DOI: 10.1021/acs.analchem.5b01988
6. Malecka, K.; Stachyra, A.; Góra-Sochacka, A.; Sirko, A.; Zagórski-Ostoja, W.; Dehaen, W.; Radecka, H.; Radecki, J. New redox-active layer create via epoxy–amine reaction – The base of genosensor for the detection of specific DNA and RNA sequences of avian influenza virus H5N1. *Biosens. Bioelectron.* **2015**, *65*, 427–434. DOI: 10.1016/j.bios.2014.10.069.
7. Gao, F.; Du, L.; Zhang, Y.; Tang, D.; Du, Y. Molecular beacon mediated circular strand displacement strategy for constructing a ratiometric electrochemical deoxyribonucleic acid sensor. *Anal. Chim. Acta* **2015**, *883*, 67-73. DOI: 10.1016/j.aca.2015.04.058.
8. Gao, F.; Fan, T.; Wu, J.; Liu, S.; Du, Y.; Yao, Y.; Zhou, F.; Zhang, Y.; Liao, X.; Geng, D. Proximity hybridization triggered hemin/G-quadruplex formation for construction a label-free and signal-on electrochemical DNA sensor. *Biosens. Bioelectron.* **2017**, *96*, 62–67. DOI: 10.1016/j.bios.2017.04.024.
9. Ye, Y.; Liu, Y.; He, S.; Xu, X.; Cao, X.; Ye, Y.; Zheng, H. Ultrasensitive electrochemical DNA sensor for virulence *invA* gene of *Salmonella* using silver nanoclusters as signal probe. *Sens. Actuators, B* **2018**, *272*, 53–59. DOI: 10.1016/j.snb.2018.05.133.
10. Senel, M; Dervisevic, M.; Kokkokoğlu, F. Electrochemical DNA biosensors for label-free breast cancer gene marker detection. *Anal. Bioanal. Chem.* **2019**, *411*, 2925-2935. DOI: 10.1007/s00216-019-01739-9.
